# Adaptive Pedestrian Stride Estimation for Localization: From Multi-Gait Perspective

**DOI:** 10.3390/s22082840

**Published:** 2022-04-07

**Authors:** Chao Huang, Fuping Zhang, Zhengyi Xu, Jianming Wei

**Affiliations:** 1 Shanghai Advanced Research Institute, Chinese Academy of Sciences, Shanghai 201210, China; huangc@sari.ac.cn (C.H.); zhangfp@sari.ac.cn (F.Z.); wjm@sari.ac.cn (J.W.); 2 School of Electronic, Electrical and Communication Engineering, University of Chinese Academy of Sciences, Beijing 100049, China

**Keywords:** inertial measurement units, indoor localization, stride length estimation, stride segmentation, gait recognition

## Abstract

Accurate and reliable stride length estimation modules play a significant role in Pedestrian Dead Reckoning (PDR) systems, but the accuracy of stride length calculation suffers from individual differences. This paper presents a stride length prediction strategy for PDR systems that can be adapted across individuals and broad walking velocity fields. It consists of a multi-gait division algorithm, which can divide a full stride into push-off, swing, heel-strike, and stance based on multi-axis IMU data. Additionally, based on the acquired gait phases, the correlation between multiple features of distinct gait phases and the stride length is analyzed, and multi regression models are merged to output the stride length value. In experimental tests, the gait segmentation algorithm provided gait phases division with the F-score of 0.811, 0.748, 0.805, and 0.819 for stance, push-off, swing, heel-strike, respectively, and IoU of 0.482, 0.69, 0.509 for push-off, swing, heel-strike, respectively. The root means square error (RMSE) of our proposed stride length estimation was 151.933, and the relative error for total distance in varying walking speed tests was less than 2%. The experimental results validated that our proposed gait phase segmentation algorithm can accurately recognize gait phases for individuals with wide walking speed ranges. With no need for parameter modification, the stride length method based on the fusion of multiple predictions from different gait phases can provide better accuracy than the estimations based on the full stride.

## 1. Introduction

Pedestrian localization is commonly used in maneuvers, fire drills, and mine rescues. Unlike GPS, optical, audio, and other sensor data, inertial measurements are infrastructure-independent, allowing them to be used for a terminal location in complex contexts [1,2]. As a result of the development of Micro-Electro-Mechanical Systems (MEMS), Inertial Measurement Units (IMUs) have become lightweight, low power consumption, low cost, and non-intrusive to users, which are suitable characteristics for clinical and residential applications. Thus, IMU-based Pedestrian Dead Reckoning (PDR) has become popular and received considerable attention [3,4,5,6,7].

Stride length estimation is one of the essential components of the PDR system [8,9,10,11,12]. There are mainly two classes of approaches: the first kind of methods are based on the integration of the accelerations, and the other techniques utilize various models to predict the stride length. The first kind of models can be further divided according to whether they are based on physical or statistical models. The double integration of acceleration in the forward direction is the most direct method for estimating stride length because it needs no assumption or user customization. However, it is not easy to obtain the forward acceleration from IMU measurements since each part of the body moves in different directions during walking [13]. Biomechanical models for step length estimating, like inverted pendulum models, are defined mainly by simplifying and approximating the mechanical movements of the human body. Nevertheless, due to the significant variability of pedestrians, these models need to be calibrated for each user. Mechanical models are also impacted by the non-negligible bias and noise of the IMU, which makes the distance error grow cubically over time or distance [14]. To reduce the cumulative error, Zero-Velocity-Update (ZUPT) was introduced to reset the integral computations for length when the foot was recognized as remaining stationary on the ground [15,16,17,18,19,20]. However, it has been found that excessive use of ZUPT can lead to a much smaller prediction result than actual walking distance. The effectiveness of ZUPT depends on the accuracy of the recognition of the zero velocity stages during foot movement. If it is too sensitive, the distance belonging to the two ends of the stride cycle will be excluded, which will be counterproductive to the stride length estimation.

To avoid the inertial drift in stride length caused by the sensors, multiple statistical variables have been found that show a clear correlation with step length and can therefore work as features or predictors in statistical models [21,22,23,24]. This type of method needs to create an empirical regression model based on the movement features of the pedestrian’s pelvis, feet, or legs and then fit the model parameters by utilizing the existing dataset to estimate the step length for walking. Li’s model demonstrates a linear relationship between step length and walking frequency [25]. Weinberg’s model utilizes the difference between the maximum and the minimum in vertical acceleration data within a step [21]. Kim’s model is only based on the mean acceleration within a step [22]. Scarlett’s model uses minimum, maximum and average acceleration to estimate step length [24]. Since the variability of individuals’ walking habits stems from gender, height, age, and walking speed, these empirical models require parameter customization for individual pedestrians. With the wide application of wearable devices, a large amount of crowdsourced data based on individual pedestrians will gradually be formed, which will bring opportunities for data-driven approaches to improve accuracy and generalization [26]. In recent years, neural networks have been developed for step length prediction [27,28]. They achieve better prediction accuracy than empirical models with the cost of larger-scale datasets and massive computation consumption. With limited training data, neural networks are prone to be overfitted. The requirement for a large number of computational resources prevents them from being used in wearable devices and embedded systems.

**Figure 1:**
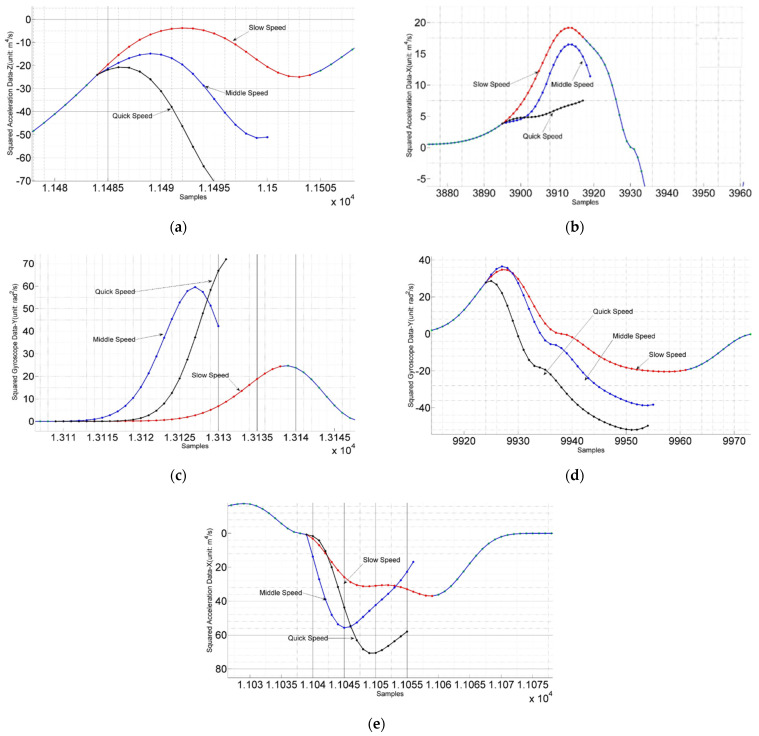
The magnitude of the sensor signal mutation at different walking speeds (**a**) The left and right direction gyroscope signals at different speeds in the stage when the foot is pushing off the ground (**b**) The acceleration signals of the front and rear directions at different speeds in the stage when the foot is pushing off the ground (**c**) In the foot swing stage, the left and right direction signals of the gyroscope at different speeds (**d**) The acceleration signal of the front and rear directions at different speeds during the heel-to-ground cushioning stage (**e**) The vertical acceleration signal at different walking speeds in the heel-to-ground cushioning stage.

**Figure 2:**
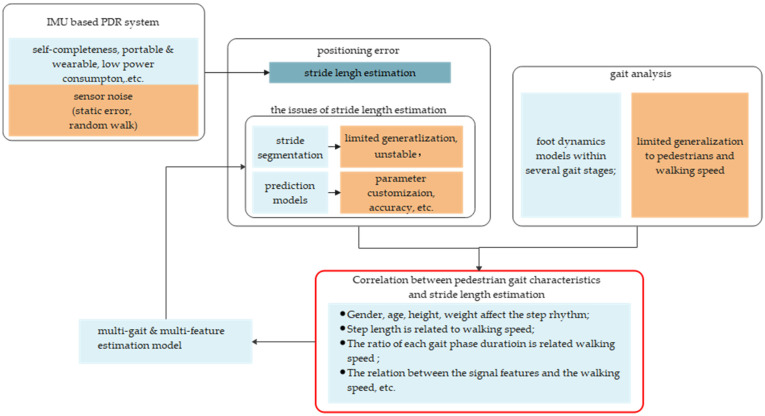
The overall idea of this article.

To summarize the preceding stride length estimation approaches, they all treat a single stride as a whole processing item rather than dealing with more detailed decomposition and analysis. Firstly, a segment of the signal corresponding to a stride must be detected. Then selected features need to be calculated and input into a pre-trained model to predict movement distance. In the field of kinesiology, IMU-based mobile gait analysis enables a continuous and detailed insight into the motor performance of foot movements in multiple gait patterns under more natural and realistic conditions compared to laboratory settings [29]. Gait characteristics are the reflection of the pedestrian’s physiological characteristics and emotions on foot movement and are closely related to the stride length. A typical phenomenon is that the fluctuation range of sensor data in the gait phase is related to the walking speed, which is shown in Figure 1, and the walking speed greatly affects stride length [30,31,32,33,34,35]. Inspired by this, we think it is possible to improve the accuracy of stride length estimation based on gait analysis. By accurately dividing a stride into several gait segments, stride length estimation can be transformed into a fusion of several predictions from different gait analyses. The outline of the ideas in this section can be seen in Figure 2.

The purpose of this paper consists of the following three parts. Firstly, we gave a detailed description of signal variation in different gait phases based on biomechanics and annotated gait phases provided by the Diverse Gait Data, which was proposed in our previous work. Secondly, we propose to divide a normal walking stride into the stance phase and three dynamic phases. Last but not least, we offer an example of the combination of stride lengths from multiple gait phases to validate the utility of gait segmentation for adaptive stride length estimation.

## 2. Materials and Methods

### 2.1. Data Foundation

The database of this part of the study is the Diverse Gait Dataset established in our previous work [26]. A total of 22 healthy volunteers (13 males, 9 females, age 32.5 ± 7.5 years) participated in the study and were divided into different groups according to gender and height information, as shown in Table 1. Each subject walked at three kinds of self-selected speeds along an indoor corridor of 46 m. The dataset contains the stride and gait information of different pedestrians walking at different speeds. The label information provides the time range of each real stride cycle and real gait stage. The stride labels and gait labels were generated based on the foot movement video while each subject was walking. Before the label is generated, the foot motion data collected by IMU has been time synchronized with the video information. The relative positions of the X,Y,Z-axis directions of the IMU module and the navigation coordinate systems are as follows: when the foot remains relatively static with the ground, the X-axis of the IMU is close to the left and right direction, and is consistent with the coronal axis in the human motion anatomical coordinate system; the Y-axis is close to the rear side of the pedestrian’s travel direction, and is consistent with the sagittal axis in the human motion anatomical coordinate system; the Z-axis is close to the ground and vertically upward, and corresponds to the vertical axis in the human motion anatomical coordinate system.

### 2.2. Adaptive Stride Segmentation Technique

Previous studies have demonstrated that stride detection is a significant prerequisite for PDR and is required to provide robust results [36,37]. In this paper, based on our previous work, the SDATW method is utilized to implement adaptive stride segmentation for different pedestrians in wide-speed domain scenarios. The SDATW algorithm has the following features. Firstly, the algorithm uses different feature extraction functions to obtain each sample point’s neighbor’s distribution coefficients and each element’s neighbor within the stride template. The feature extraction functions include calculating the signal amplitude distribution, the average gradient change of the signal, or the wavelet decomposition coefficients of the signal in the neighborhood.

By extracting the features of each sampling point, the influence of sensor noise can be reduced, and the representative components within the local area of the signal can be acquired. We could obtain a smooth cumulative distance function benefit from this advantage. In addition, SDATW differs from traditional DTW in finding the optimal alignments [38,39]. The conventional methods regenerate a sliding window for each sample point and calculate the cumulative distance between the sliding window and the template, which seems to be a heavy computational burden. SDATW only needs to calculate the accumulated difference between two neighborhoods of the sample point and the template’s element, whose size is much smaller than a sliding window. Compared with the traditional DTW methods, it is less computationally intensive and, therefore, can work with less requirement for computational resources. Last but least, the SDATW algorithm obtains the best matching segment with the template while comparing the accumulated distance corresponding to the starting and ending position of the data segment with all competing points. In the process of finding the optimal alignment path, the path with minimum accumulated distance and its corresponding beginning point are recorded simultaneously. The subsequence in the continuous data stream that best matches the standard stride template is dynamically obtained. The beginning points and the ending points are recognized as the boundaries of the stride. The best match segment is found by comparing its starting point and ending point with local competitors, so SDATW is independent of thresholds compared with previous methods. When used for stride detection in cross-individual and broad speed domain scenarios, SDATW does not require parameter modification and has better robustness to changes in movement habits and walking speed of different individuals. We refer the interested readers for a more detailed calculation process [40]. The standard stride template is constructed based on 1407 strides data interpolated or down-sampled to form a stride database matrix in which each line of the sequence is fixed with the same length. And then, the template is obtained by calculating the average value in each column. These strides correspond to various pedestrian subjects with different heights, different genders, and various walking speed fields. All these data were randomly selected by 30 percent from the Diverse Gait Dataset collected in our previous work.

**Figure 3:**
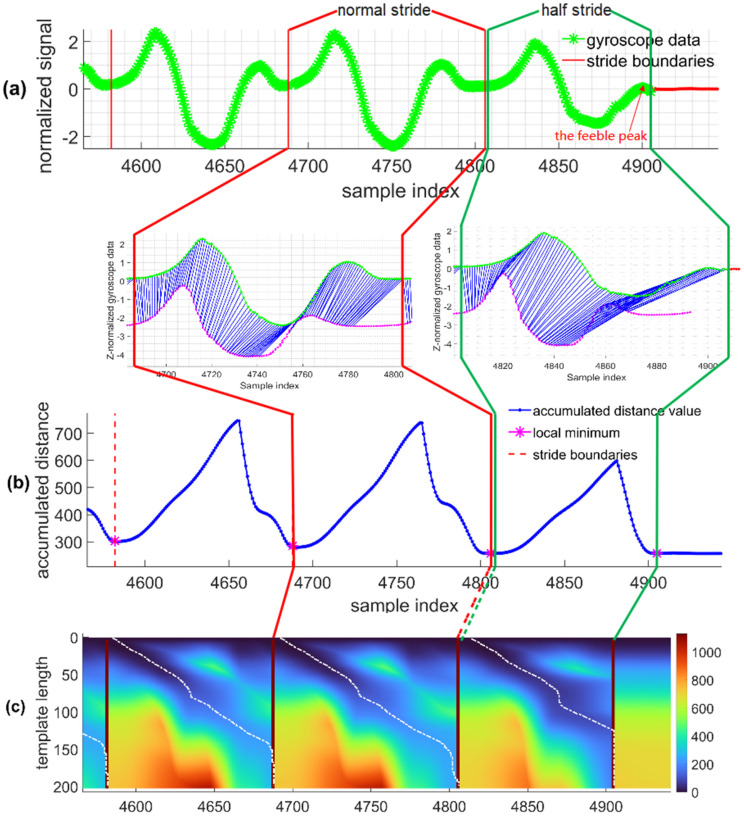
The effect of stride segmentation method based on SDATW algorithm. (**a**) After applying the SDATW on gyroscope coronal-axis data, the stride borders are available, which are represented as red vertical lines. And the stride segments just look like the template. Two subfigures demonstrate detailed alignment pairs of the two segments corresponding to the stride template; (**b**) The accumulated distance curve used in SDATW methods maintain the smoothness and monotonicity in normal strides and the “half-stride” situations; (**c**) Three white lines represent the warping paths which correspond to best-matched subsequences in the query sequence. Dark red ribbons between two warping paths indicate the borders of detected stride segments. The red lines and the dark green lines are used to show related information of a normal stride and a half stride, respectively.

As demonstrated in Figure 3a, a normal stride is marked as stride (1) (S1), and a ‘half-stride’ is marked as stride (2) (S2). The periodic length between the S1 and S2 seems not apparent, but S2 is the last stride of a continuous walk while S1 is one of the normal strides. During S2, the subject walked and covered just half of his regular stride length and then landed the foot on the ground. It can be shown that the signal in S2 in Figure 3a is generally smaller in magnitude compared to its last stride and just forms a feeble peak followed by the curve close to zero value (annotated by a red arrow). This reflects the movement process that the last part of S2 ended swiftly and then kept static with the ground, so we call it the “half-stride”. The “half-strides” exist commonly in normal pedestrian walking and are easily dismissed in odometers. Although the error is acceptable for step counting, it impacts the accuracy of stride length estimation, so we need to ensure that “half-stride” should be correctly identified. As shown in Figure 3b, even S2 is different from the usual cases, the accumulated distance curve of SDATW remains smooth and consistently monotonic and identifies the boundaries of the S2. The two subplots below Figure 3a show detailed alignment pairs of the two segments corresponding to the strides with the template, respectively. It can be visually noticed from the comparison of the two subplots that the proportion of matched points between S2 and the stride template is smaller than that between S1 and the template. In Figure 3c, three white curves run through the accumulated distance matrix colormap and represent the warping paths that correspond to best-matched subsequences in the sensor signal. We can find that the warping course of S2 runs close to the right boundary earlier than S1’s warping path, indicating that there is less matching part with the stride template, which is consistent with subfigures in Figure 3a.

### 2.3. Stride Gait Division Method

Based on the results of stride segmentation, we were able to divide the IMU data of a regular walking stride into four segments with motion semantics: {push-off, swing, heel-strike, stance} within a plausible time segment. In the following, we will first present the basis for the recognizing gait phases, followed by the data preprocessing procedure. We will implement the gait segmentation method based on the acceleration in the anterior-posterior direction and the vertical movement of the ground based on the prepared data.

#### 2.3.1. The Understanding of Gait Modes

Understanding foot movement data is the basis and foundation for the analysis of gait segmentation and gait characterization. Although this does not involve subtle algorithms or models, we believe that understanding motion data in this section is essential for the clarity and accuracy of our proposed gait segmentation method.

The database for this part of the study is the Diverse Gait Dataset, which contains IMU recordings and time information of strides and gait phases from different pedestrians in various walking speed states, where the labels provide the boundaries of the complete stride on the time axis, as well as the temporal boundaries of each gait phase. The sampling frequency of the video recording is a minimum of 60 Hz and a maximum of 240 Hz, in which the motion of the foot during a complete stride cycle is presented.

**Figure 4:**
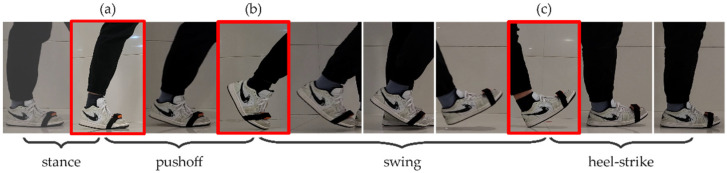
The movement of a whole stride can be divided into four gait phases. (**a**) displays that the heel just leaves the ground at the end of stance phase; (**b**) displays that the toe is going to leave the ground at the end of push-off phase; (**c**) displays that the heel touches the ground at the end of swing phase.

With reference to video recordings, we can visually divide a full stride into three dynamic phases with distinctive features and one stationary phase, as shown in Figure 4 [40]. When the toe leaves the ground contact, it is considered the push-off. If the heel starts to contact the ground, it indicates the beginning of the heel-stride. When the heel starts phase’s end to leave the ground, that can be comprehended as the end of the stance. For two adjacent gait phases in the sequence, the endpoint of the previous phase can also be considered as the starting point of the next phase.

The stationary phase, in which the foot remains relatively static with respect to the ground, does not contain any dynamic information and is therefore not analyzed. Then the three dynamical gait phases are combined with the video information to analyze the corresponding acceleration data and gyroscope data. This part is the basis of our direct segmentation of the sensor data into different dynamic phases without video information.

The accelerometer records the force of the support surface to which the inertial device is subjected. When the foot is at rest relative to the ground, the module is balanced by gravity and the support force of the land. The accelerometer Y-axis shown in the figure is close to the vertical direction of the ground, with a numerical magnitude of approximately the acceleration of gravity, and the value of the X-axis is close to 0.

**Figure 5:**
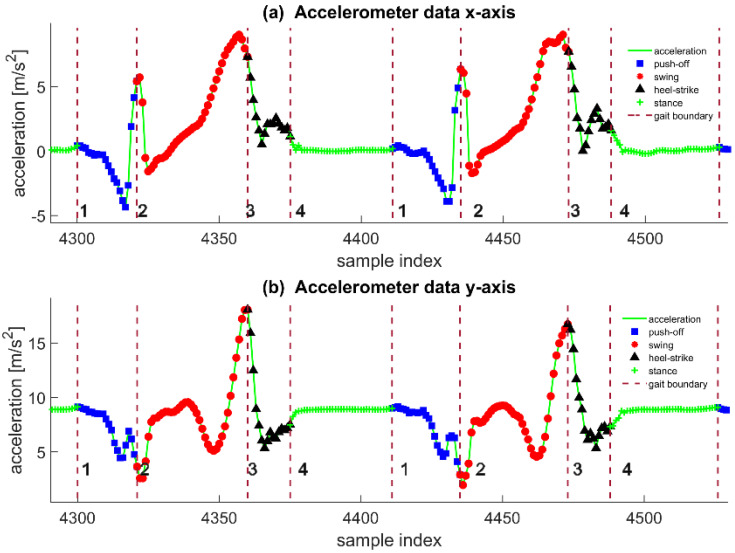
Gait segmentation label display. (**a**) Annotated gait phases in sagittal acceleration data; (**b**) Annotated gait phases in vertical acceleration data.

The push-off phase is coming behind the stance phase. During this phase, the height of the heel gradually increases as the ankle begins to rotate, and the bottom of the foot gradually leaves the ground. In general, since the mobility of the ankle and toe joints varies from person to person and the angle and speed of rotation of the foot around the Z-axis at different walking speeds, the push-off phase data contains information on walking speed individual walking habits of the pedestrian. However, during normal walking, the foot movement in the push-off phase has this trend. While the XOY plane of the IMU module is rotating around the Z-axis and the angle with the ground gradually increases, the X-axis gradually turns and tends to the vertical direction of the ground. The Y-axis gradually turns and tends towards the forward direction. Band 1 in Figure 5 shows the process of X-axis and Y-axis signal changes. During this stage, the X-axis value of zero in the static phase gradually decreases to approximately equal to the negative gravitational acceleration because the positive direction of the X-axis is almost perpendicular to the ground and pointing downward. In contrast, the support force of the land is upward at this time, precisely opposite to the positive direction of the X-axis. The value of the Y-axis gradually decreases from close to the gravitational acceleration in the static phase to zero.

At the end of the push-off phase, only the toe is in contact with the ground, and the angle between the foot and the ground reaches the maximum value of the entire process. The force accumulated in the whole foot is about to explode at the toe and produce a sudden change of force. When the toe leaves the ground, it moves upward in an oblique direction. During this short process, the change of the signal in terms of X-axis acceleration is noticeable, which is expressed as a monotonous and sharp ascending segment, so the effect of the force generated at this time is in the X-axis and Y-axis, opposite to the first half of band1, which also leads to the fact that between band 1 and band 2, the X-axis and Y-axis acceleration data produce their respective peaks and valleys.

In the swing phase, the foot swings in the air until the heel makes contact with the ground. At the same time, the foot still rotates around the X-axis; unlike push-off, the height of the toe gradually rises, the XOY plane of the IMU module revolves around the Z-axis, and the Y-axis starts from the angle of vertical ground upward gradually rotates to the forward direction. It then continues to move diagonally upward toward the ground. Then the foot will gradually approach the ground. In the final stage of the swing, the foot does not glide on the floor but produces a significant impact at the moment of contact between the heel and the ground. The friction generated by this considerable interaction force makes the heel immediately finish swinging state.

Observing the synchronized acceleration data, we can achieve a more detailed description. At the beginning of the swing, the force with the ground disappears immediately because the interaction force disappears immediately when the foot leaves the ground. This is reflected at the beginning of band 2 in Figure 5. The X-axis acceleration decreases sharply, and the Y-axis acceleration quickly returns to a level close to gravitational acceleration. At the end of the swing phase, the heel first approaches and hits the ground, thus generating an interaction force much larger than the gravitational acceleration. We can see in Figure 5b that the Y-axis acceleration starts to decrease in the middle of band 2. Then the acceleration at the end of band 2 quickly rises to a peak, which corresponds to the downward motion of the heel approaching the ground first and the reaction force of the ground after the impact. In Figure 5a, the sample point in X-axis acceleration is not at the peak at the end of band 2. This is because the toe is still maintaining the motion inertia. Still, since the heel is already locked to the ground, the toe can only make a rotational motion, in which the forward acceleration exists but has shown a tendency to decay. This process causes a second abrupt change in the force of the foot in completing the stride motion. This abrupt change causes a distinct peak in the Y-axis data, and the peak in the X-axis is similar in time to the peak in the Y-axis data.

The heel-strike phase is a short deceleration cushion phase for the foot. This process is reproduced from the typical characteristics of pedestrian walking: the foot rotates downward on the axis of the heel, with the toe at the end of the foot, reflecting the most evident speed change. During this phase, the toe is subjected to a significant deceleration. Therefore, it can be seen in band 3 of Figure 5a that the X-axis acceleration is decreasing rapidly from the beginning of the heel-strike. In band 3 of Figure 5b, the Y-axis acceleration also decreases. This is because the impact force with the ground is transferred to the arch and the leg; the ground reaction force is reduced and gradually converges to the ground support force on foot. In the later stage of the heel-strike, the forefoot touches the ground. It makes a “slap on the ground” movement, which results in the foot receiving an upward ground reaction force in the vertical direction and a side effect in the forward direction, where the foot again receives a ground reaction force. We find a sub-peak point in the subjects’ walking data sequence in the second half of band 3 in Figure 5b. Afterward, since the Y- and Z-axis data decrease with the same trend to a relatively constant value, this means that the IMU module that follows the foot movement also enters a temporary stationary phase.

Based on the above description of the foot motion, we interpret the correspondence between the gait boundaries and the acceleration peaks or valleys in the anterior-posterior and vertical directions. Therefore, in this paper, we propose a gait segmentation method based on signal time-domain analysis and use this method to realize and validate our interpretation.

#### 2.3.2. Recognizing Gait Boundaries Using Peak-Valley-Pairs

Based on the understanding of foot motion in different gait phases in this paper, we will analyze the peak and valley values of acceleration at two gait boundaries, which correspond to two abrupt changes in the foot-ground interaction forces in a complete stride cycle. Among the sensor data, the acceleration data record the change in the force between the foot and the ground, so it is more suitable for delineating the gait pattern. The foot’s motion reflects periodic forces acting mainly in the anterior-posterior and vertical directions when pedestrians walk. At the same time, it is susceptible to individual differences in the coronal axis (X), so we choose to implement gait classification based on sagittal (Y) and vertical axis (Z) acceleration data. Since the peaks and valleys we want to analyze are between the stance phases, we choose to detect the stance gait phase within a period of the stride cycle first and then divide the remaining three dynamic phases.

The most apparent difference between the stance phase and the other three dynamic phases is that in this phase, the foot remains relatively stationary with the ground, so the amplitude of the acceleration data is much smaller than in the other phases. In order to more clearly reflect the difference between the amplitude of the acceleration data in the stance phase and the other part, we first filtered out the high-frequency noise in the signal using a mean filter with a fixed sliding window. Then we deflate the signal by first squaring the original signal and then multiplying it by its original sign. This increases the amplitude of the acceleration signal in dynamical phases and makes the signal of the stance phase closer to zero, so we can easily identify the signal segment corresponding to stance phases. By calculating the volatility for the pre-processed signal and then using a thresholding method, we can detect the static phase in the stride cycle. In this paper, variance and interquartile range (IQR) are used to measure the volatility of the acceleration along the vertical axis and sagittal axis, and the data segments in the stride cycle where the variance and quadrature difference are less than their respective thresholds are judged as static phases.

By understanding the signal in Section 2.3.1, we know that during forward walking, the foot is swinging forward by inertia during the swing phase, which means that there is no additional force on the foot during the swing phase. Our hypothesis is also corroborated by the observation of the acceleration differential signal. As shown in the Figure 5, the acceleration differential signals in both X and Y axes show a “static” characteristic in the swing phase, while several peaks and valleys appear in the push-off and heel-strike phases, which reflect the variation of the foot force on the ground.

This gives us a periodic distribution of peak and trough points on the time axis: between the two stance gaits (the former stance phase comes from the previous stride, the later stance phase belongs to the current stride), the abrupt changes in foot-ground interaction forces are concentrated in two clusters, while between the clusters they are in a relatively stable segment of the relative foot forces, with very few sharp peaks or valleys. Based on this observation, we use the k-means clustering algorithm (k = 2) to divide the peak and valley points of vertical-axis and sagittal-axis acceleration signals into two classes, and the clustering results, the left cluster corresponds to the peak and valley values in the push-off phase, and the right cluster corresponds to the peak and valley values in the heel-strike phase. The clustering results allow us to determine the boundary points of the two gait phases, push-off/swing and swing/heel-strike, in a smaller and more certain time range. If we can further determine these two boundary points, then the 3 dynamic phases of a complete stride cycle are cut apart. The pseudo-code of the Algorithm 1 is as follows.
**Algorithm 1:** Peak-points clustering pseudo code**Input:** [n_ last_stance_end, n_current_stance_beginning, ary_peaks]**Output:** [left_peaks, right_peaks}**Initialize:** center_0 = n_ last_stance_end, center_1 = n_current_stance_beginning, center_0_old = inf, center_1_old = inf, iter_num = 0;**1**while iter_num < 50 do**2** iter_num++;**3** for point in ary_peaks do**4**  if $|\left|\mathrm{point}\_\mathrm{index}-\mathrm{center}\_0\right|⬚{\left.\right|}_{2}<{\left|\left|\mathrm{point}\_\mathrm{index}-\mathrm{center}\_1\right|\right|}_{2}$
**5**    left_peaks = [left_peaks; point_index ];**6**  else**7**    right_peaks = [right_peaks; point_index ];**8**  end**9** center_0_old = center_0, center_1_old = center_1;**10** center_0 = mean(left_peaks), center_1 = mean(right_peaks);**11** if $|\left|\mathrm{center}\_0-\mathrm{center}\_0\_\mathrm{old}\;\right|⬚{\left.\right|}_{2}<\varepsilon$ and $|\left|\mathrm{center}\_1-\mathrm{center}\_1\_\mathrm{old}\;\right|⬚{\left.\right|}_{2}<\varepsilon$
**12**  break;**13** else**14**  continue;**15** end**16** **return** [left_peaks, right_peaks]

Based on our understanding of acceleration data and the available gait labeling information, we find that acceleration data near the boundary of the dynamic gait varies very dramatically compared to the surrounding data, which is usually manifested by the signal rapidly crossing the zero axis negatively from a towering peak and decaying to a trough, or vice versa. This variation makes these peaks and trough points very different from other peaks or troughs in their neighborhood, and they all necessarily cross the zero axis with the distance between the peak and trough much greater than the amplitude of the other peaks or troughs. In addition, during normal walking, the foot moves continuously and smoothly during a stride cycle rather than mixed with actions such as secondary starts, so there is often only one pair of these prominent peaks and valleys. Even though we were able to find the valley closest to it in time for each peak, the difference between the peak and the valley was more significant the closer we got to the gait boundary. Based on this observation, we designed the “major peak-valley-pair” as the object of querying the gait boundary. The closest valley in time is found backward as a “peak-valley pair” for each peak. To find the “major peak-valley pair,” we add several conditions: (1) the peak and the valley must contain 0 crossing between them; (2) the magnitude between the peak and the valley is always the largest; (3) if there is a “peak-valley pair,” the conditions (1) and (2) are satisfied, the distance between the two points is greater than 1/2 of the distance of the “major peak-valley pair,” and the difference in position between the peak and the “prominent valley” is less than five samples, then the two pairs of “peak-valley pairs” are combined into a new “major peak-valley pair.” Condition (3) is based on our practical experience, making the “major peak-valley pair” more stable when encountering noisy acceleration sequences.

When finding the swing/heel-strike gait boundary, we determine the position of the peak point of the “major peak-valley pair” on the sagittal acceleration and vertical acceleration in the time axis as the gait boundary, and take the average value as the final result; when finding the push-off/swing gait boundary, we determine the position of the peak point of the “major peak-valley pair” on the sagittal axis and the valley point of the “major peak-valley pair” on the vertical axis as the gait boundary and take the average value as the final result. In finding the push-off/swing gait boundary, we determine the position of the peak point of the “major peak-valley pair” on the sagittal -axis and the position of the valuation point of the “major peak-valley pair” on the vertical axis in the time axis as the gait boundary, and take the average value as the final result.

**Figure 6:**
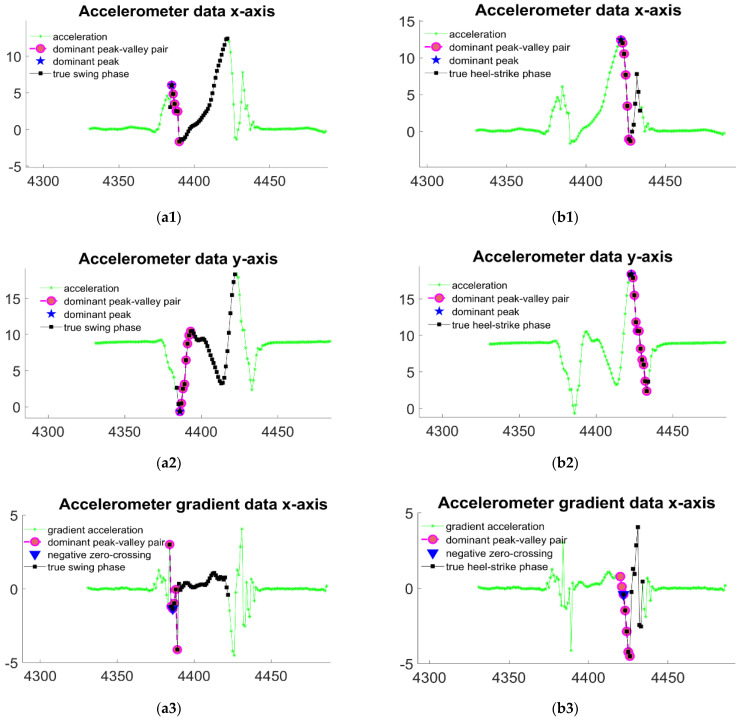

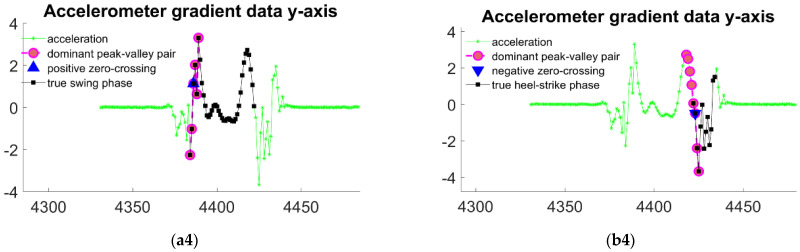
The detection of boundaries between push-off phases and swing phases are shown in subfigures (**a1**–**a4**). The boundaries for dividing swing phases and heel-strike phases are shown in subfigures (**b1**–**b4**). Among all subfigures, (**a1**,**b1**) both use major peak-valley pairs in sagittal acceleration as reference information; (**a2**,**b2**) both use major peak-valley pairs in vertical acceleration as reference information; (**a3**,**b3**) show the utility of zero-crossings in sagittal acceleration data as reference information; (**a4**,**b4**) show the utility of zero-crossings in vertical acceleration data as reference information to recognize the gait boundaries.

Furthermore, we also consider the derivatives of the sagittal acceleration and vertical acceleration data as a reference for determining the gait boundaries since the peak points of the valley points in the signal correspond to its derivative sequence’s zero-crossing points. The positive crossings correspond to the valley points, and the negative crossings correspond to the peak points. Therefore, under ideal circumstances, the positive zero-axis crossing point in the derivative sequence of the original signal should share the same timestamp as the valley point used to determine the gait boundary, which can be demonstrated by comparing Figure 6(a2,a4). Moreover, the timestamp of the negative zeros-axis crossing point in the derivative sequence should be equal to that of the peak point used for the gait boundary decision.

Therefore, we also provide an augmented version of the gait segmentation algorithm. In finding the swing/heel-strike gait boundary, we consider the timestamps of the two zero-axis negative crossing points in the derivative sequence of the sagittal and vertical axes, which are nearest to the “major peak-valley pair”, as another two references. For the decision of push-off/swing gait boundaries, we select the negative zero-axis crossing point with the least time difference to the “major peak-valley pair” in the sagittal acceleration signal, and the positive zero-axis crossing point with the least time difference to the “major peak-valley pair” in vertical acceleration signal as two more bases for determination. Before calculating the mean value to estimate the gait boundary, we also performed an outlier detection on the four reference items. The process of outlier detection can be simply stated as follows: we use a “leave-one-out” approach to fit an approximately normal distribution to the remaining points in the set and obtain the expected value and standard deviation. If the Euclidean distance between the left-out point and the expected value is greater than three times the standard deviation, the point should be eliminated as an outlier. Then the value of the remaining valid reference will be averaged to find the estimate of gait boundary location.

### 2.4. Adaptive Stride Length Estimation

#### 2.4.1. Dataset for Training Stride Length Estimation Models

The training of regression models for stride length estimation requires datasets that can provide accurate distance measurements. We selected a high precision reference dataset for pedestrian navigation proposed by researchers from the German Aerospace Center (DLR) [28] in this part of the work. The dataset is applicable to analyzing a broad range of indoor positioning approaches. The reference information was collected by employing a high-precision optical reference system that could pinpoint the marker mounted on the pedestrian’s shoe within a millimeter at a rate of about 100 Hz. The foot movement data was recorded using a foot-mounted IMU with a sampling frequency of 100 Hz. The dataset consists of recordings while several pedestrians were walking, running, and backward walking. Since running gait and backward walking gait are out of the scope of this paper, we selected eleven of sixteen files with a total of 231 waking strides. Then the proposed gait segmentation method was applied to obtain push-off, swing and heel-strike phases, each kind of gait segment has the same volume as the strides. We will describe the process of manufacturing the models and training based on gait phases in the following sections.

#### 2.4.2. Data Preprocessing and Feature Selection

Based on the experiences models proposed by Weinberg [21], Kim [22], Scarlette [41], and Ladetto [23], we extracted the features from vertical acceleration data, sagittal acceleration data, and the mode values of the three-axis acceleration, respectively. We put them together to form the feature set for training models. There are twelve features, which include the extreme value, the average, the signal range, the signal variance, and the frequency corresponding to the gait segment. We list them in Table 2, where $a_{max}$ and $a_{min}$ represent the maximum and minimum of the acceleration sequence, $\left|a\right|$ represents the absolute value of each sample, f represents the frequency, and v represents the variance of a sequence of acceleration data.

In this literature, we also use standardization, which is necessary for the accuracy and generalization of regression models. It refers to the process of normalizing every sample in a series of data such that the mean of all values is 0 and the standard deviation is 1. Hence, it helps to relieve the impact of outlier points on stride length estimation results. The calculation is shown in the following formula:

(5) $$acc_{norm,i}=\frac{acc_{i}-\bar{acc}}{S},\;i=1,2,\ldots ,n$$

where $\bar{acc}$ is the mean value of accelerometer series, and S is the unbiased estimator of the standard deviation:

(6) $$S=\frac{1}{n-1}\;\overset{n}{\underset{i=1}{\sum }}{\left(acc_{i}-\bar{acc}\right)}^{2}$$

**Figure 7:**
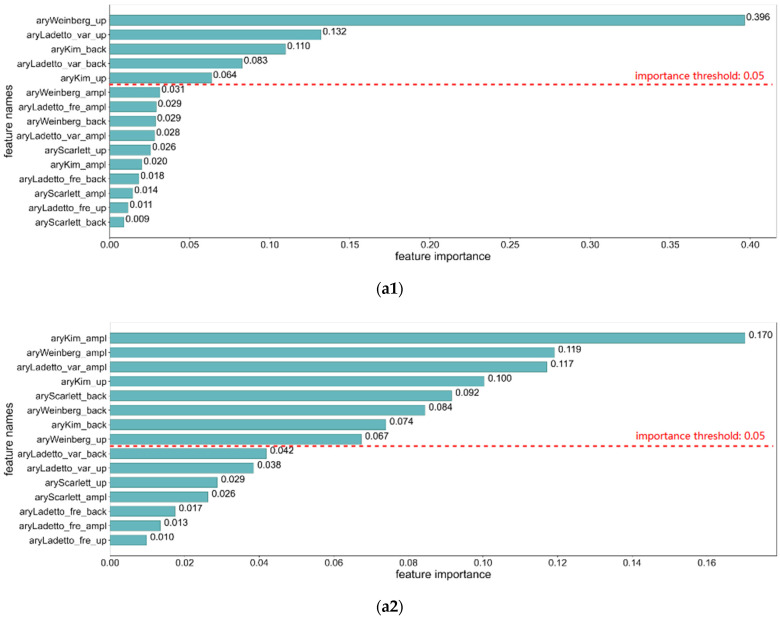

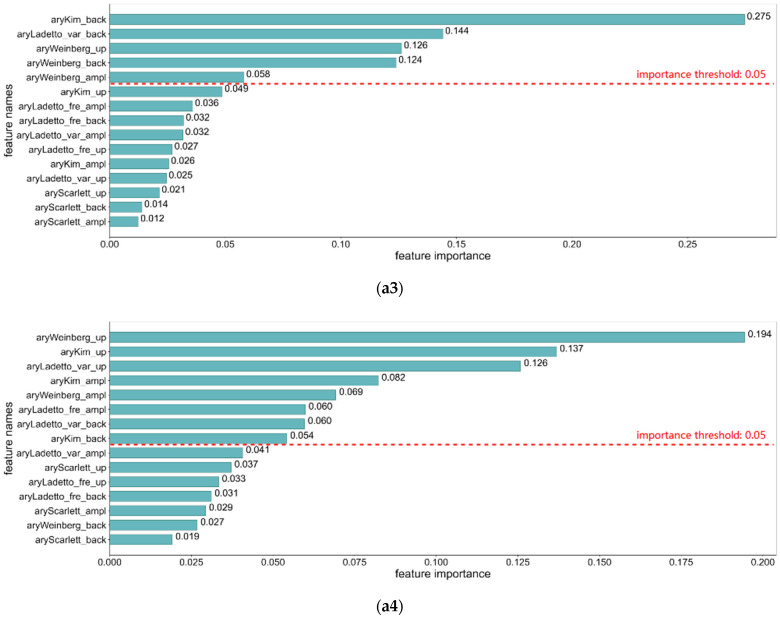
Feature importance rankings. (**a1**) Features extracted from whole stride acceleration data; (**a2**) Features extracted from push-off phase acceleration data; (**a3**) Features extracted from swing phase acceleration data; (**a4**) Features extracted from heel-strike phase acceleration data.

Based on our understanding of foot motion within different gait phases, we argue that the correlation between each feature and stride length varies across gait phases. Therefore, to prevent model overfitting, we need to reduce the dimensionality of the feature sets for training. In this part of the work, we utilize the Random Forest Regressor [42,43] function in scikit-learn to measure the importance of each feature to the stride length estimation, and the feature importance measurements in the whole stride cycle and three different gait phases are shown in Figure 7 [44,45].

It can be seen from the figure that the features are ordered by their importance values within each gait phase, and an importance threshold was set to select the features whose importance is larger than the value. During the push-off phase shown in Figure 7(a2), the number of selected features based on acceleration modulus, vertical axis acceleration, and sagittal acceleration is balanced because, during the push-off stage, the foot is angled to the ground and subjected to the forces in both vertical and sagittal axes. During the swing phase, the selected features based on sagittal axis acceleration mainly form the chosen feature set. The importance value of Kim’s element from sagittal acceleration is much larger than that of the features from the vertical axis. Because there is hardly any interaction between the foot and the ground when the foot is swinging in the air, the forces are evident in the anterior-posterior direction of walking. Within the heel-strike phase, the features based on vertical axis acceleration are the most numerous and occupy the most significant importance measurements because of the violent impact generated when the foot comes in contact with the ground. According to our analysis of foot motion, the process of the swing phases contributes the most significant part of the stride length. However, we can learn by comparing the feature ordering in full stride with that of the swing phase that the ordering is significantly different, although the main features are roughly the same. In summary, the feature ranking for the full stride and the three different gait phases shows that each gait phase contains its own way to affect the stride length so that we can train completely different stride estimation models for each phase.

#### 2.4.3. Stride Length Estimation Models

Different sets of features have been prepared for different gait stages based on the feature selection. In this part, we trained two regression models: SVM regression model with RBF as the kernel function [46], and an ensemble method in which SVM-RBF is considered as the base model in the AdaBoost algorithm [47]. We then merge the three predicted stride length values stemming from three gait stages and find the stride length estimations for the stride cycle. We will evaluate the performance of the stride length estimation models in Section 3.3.

### 2.5. Metrics for Gait Phases Division and Step Length Estimation

The error in gait phase division arises from unrecognized gait phases and pseudo-gaits. The algorithm proposed in this literature relies on extracting the main peak points and valley points in the data stream based on the threshold. However, the noise in the sensor signal and the uncertainty of the pedestrians’ foot movements while walking can make the threshold sensitive to redundant spot points, which results in the peak and valley pairs and leads to pseudo-gait phases or the missing of real gait segments. We use True Positive (TP) to represent true gaits that should be identified, False Negative (FN) to represent the true gait phases that should have been recognized, and False Positive (FP) to represent the pseudo gaits that do not correspond to any actual gait label. Precision describes the percentage of true gait phases identified by the gait segmentation algorithm among all outputs and is shown in Equation (7) [32]. Recall, as shown in Equation (8), calculates the percentage of how many items from all labeled gait phases are recognized by the gait segmentation algorithm [33]. In this paper, we use the F-score, as shown in Equation (9), which is the harmonic mean of precision and recall [34]. The highest possible value of the F-score is 1.0, indicating perfect precision and recall. That means the gait phase division method not only recognized all true gait phases with no missing but also provided no pseudo gait phases in the outputs. The lowest value of the F-score is 0 since both accuracy and recall are 0. This means that there is no real gait phase in the output of the gait segmentation algorithm, and all outputs are pseudo gait phases.

(7) $$precision=\frac{true\;positives}{true\;positives+false\;positives}$$

(8) $$recall=\frac{true\;positive}{true\;positives+fasle\;negagives}$$

(9) $$F–score=2\times \frac{precision\times recall}{precision+recall}$$

In addition, we consider that a proper gait segmentation method should not only find out all real gait phases and avoid pseudo-gait phases but also need to ensure that the detected gait phases are as close as possible to the time range of the real gait. F-score only indicates whether the signal segment from the output corresponds to the real gait phase, but it does not measure similarity between the data segment corresponding to the output and that corresponding to the real gait from the dataset. Here we use Intersection over Union (IoU) as the other metric for the gait phase segmentation algorithm. IoU is a term used to describe the extent of overlap of two boxes in object detection problems in the computer vision area [48]. The detection method aims to keep improving its performance until the predicted area as a bounded box perfectly overlaps with an annotated target area in another box. To formally apply IoU to evaluate a gait phase detector, we need the time range of detected gait and that corresponding to the real gait signal segment. As long as we have these two sets of bounded time segments, we can apply IoU in our circumstances.

Let us assume that the time range of segment A is represented by $\left[t_{p0},t_{p1},\ldots ,t_{pm}\right]$, the time range of segment B is presented by $\left[t_{q0},t_{q1},\ldots ,t_{qn}\right]$, where m and n are the lengths of the segment A and the segment B, respectively. In addition, that the elements of the time range are time stamps of sampled data or the index value in the whole signal data stream. If there exists the segment $\left[t_{pa},\;\ldots t_{pb}\right],a\geq 0,b\leq m,a<b$ in the time range of segment A and the segment $\left[t_{qc},\;\ldots ,t_{qd}\right],c\geq 0,\;d\leq m,c<d$ in the time range of segment B, whose lengths are equal and share all elements between each other, i.e., the timestamps or index values, then we call $\left[t_{pa},\;\ldots t_{pb}\right]$ and $\left[t_{qc},\;\ldots ,t_{qd}\right]$ as the intersection of segment A and segment B. For the union of the two segments, we take the smaller value of $t_{p0}$ and $t_{q0}$ as the beginning and the larger value of $t_{pm}$ and $t_{qn}$ as the endpoint. Then the IoU is applied to measure the similarity between the detected gait segment, and the annotated gait segment is calculated as Equations (10)–(13):

(10) $$IoU=\frac{length\_of\_intersection}{length\_of\_union}\;$$

(11) $$length\_of\_intersection=t_{pb}-t_{pa}$$

(12) $$length\_of\_union=t_{max}-t_{min}$$

(13) $$t_{max}=\max\left(t_{qn},\;t_{pm}\right),\;t_{min}=min\left(t_{q0},t_{p0}\right)$$

For the results of stride length estimation, errors are the differences between the predicted values and the actual values of each stride. They are calculated as follows:

(14) $$\mathrm{RMSE}=\sqrt{\frac{\sum_{i=1}^{n}{\left(y_{i}-y_{p}\right)}^{2}}{n}}\;$$

where $y_{i}$ represents the value provided by reference information, $y_{p}$ represents the predicted value and n stands for the number of strides [49]. We also use relative error rate (RE) to describe how accurate the accumulated estimated distance is compared to the true distance of a sequence of continuous walking data. In most application scenarios, it is impractical to obtain the true value of each stride while a pedestrian is walking. Still, the distance of a section of the marked path could be available by means of a portable laser rangefinder. Thus, we can utilize the relative error rate to measure the difference between the true distance and the cumulative distance obtained from the predicted values of the stride estimation algorithm. The formula is shown as follows:

(15) $$\mathrm{RE}=\frac{\left|distance-\sum_{i=1}^{n}y_{i}\right|}{distance}\times 100\%$$

where distance represents the true distance value of a section of marked path, the sum of $y_{i}$ represents the accumulated stride length results.

## 3. Experiments and Results

**Figure 8:**
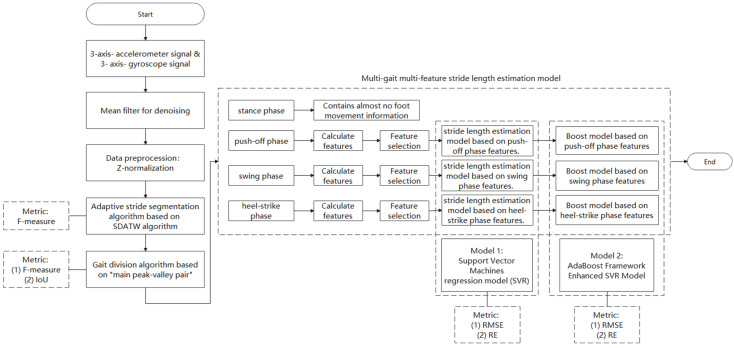
System frame diagram.

The evaluation of our proposed gait segmentation method was performed in three steps. Firstly, the adaptive stride segmentation algorithm based on SDATW is implemented on the Diverse Gait Dataset. Secondly, we evaluated the gait segmentation method based on sagittal (Y) and vertical axis (Z) acceleration data and tested it on the Diverse Gait Dataset. An augmented version of the gait segmentation method was performed, which was based on sagittal (Y) axis acceleration, vertical axis (Z) acceleration data, and their gradients, respectively; we also implemented the same test for the augmented gait segmentation algorithm and compared the F-score and IoU between two kinds of our gait segmentation algorithms. The results of our methodology were compared to two existing gait events detection algorithms based on well-established gait analysis and are suitable for foot movement data. In the third subsection, the performance of the proposed adaptive stride length estimation method is given. These three parts of the test are included in the system flow chart in Figure 8. The test results of each part use their own metric to measure the algorithm performance.

### 3.1. Performance of Stride Segmentation Algorithm Based on SDATW

To validate the utility of SDATW for stride detection, we tested the performance of the SDATW algorithm on the Diverse Gait Data. We compared it with the well-established zero-velocity detection algorithm for foot-mounted sensors and msDTW, which is chosen as a representative of the conventional DTW algorithm. We learned from Barth’s work [8] that F-score is the harmonic combination of precision and recall, and it takes into account missing strides and pseudo-strides equally. A qualified stride segmentation should perform with F-measure as close to 1 as possible. According to gait analysis in [50], walking speed dominates in influencing gait parameters over gender, age, body height, and weight. Therefore, we consider walking speed type as the critical component in the test scenario to verify the stability of our stride segmentation method. The F-score of SDATW and the other two ways are shown in Table 3. For each group of the speed-type test, we mixed foot movement data from all subjects, which brought the diversity of gender and height. In the group test named ‘all’, the strides cover three walking speed types by different male and female pedestrians with a height range of [165 cm, 190 cm], so it is close to the actual application scenarios where the stride detection methods work with no prior knowledge, and the walking speed is not limited. SDATW provides a better stride identification metric than the other two algorithms in three distinct speed groups and full-speed range tests. Therefore, using SDATW as the stride detection method in this literature can provide an accurate and trustworthy basis for stride length estimation in cross-individual and wide-speed domain scenarios.

### 3.2. Separate Performance of Gait Segmentation Algorithms Using Different Reference Schemes

The goal of this portion of the experiment is to implement and validate two versions of pedestrian walking gait segmentation algorithms under cross-individual and broad speed domain circumstances. Therefore, when preparing the experimental data, we presume that the algorithm should not have access to certain prior information aids, such as the physiological features of the pedestrians. We consider walking speed type as the critical component in the test scenario to verify the stability of our gait event detection method. For each group of the speed-type test, various pedestrian’s foot movement data are included, which brought the diversity of genders and height differences. We test the gait segmentation algorithm using vertical and sagittal axis acceleration data. We also obtained results for the other version of implementation using vertical and sagittal axis acceleration data and their derivative sequences under the same test groups.

**Figure 9:**
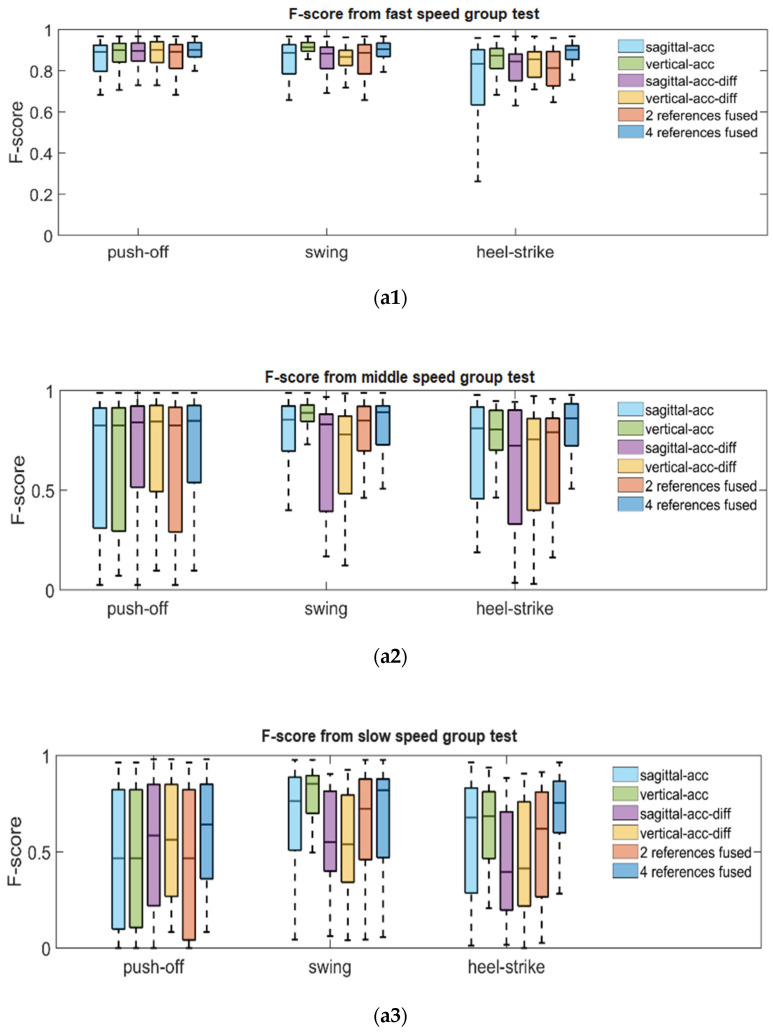
Use F-score as metric to measure the performance of gait segmentation methods for recognizing push-off phases, swing phases and heel-strike phases. Subfigures (**a1**–**a3**) shows the F-score corresponding to three gait phases in walking with fast speed, middle speed and slow speed respectively. The first four items in legend box indicate using only one reference information to detect gait phases boundaries. The ‘2 references fused’ item indicates calculating the average value between ‘sagittal-acc’ and ‘vertical-acc’ as gait boundaries. The ‘4 references fused’ item means the detected boundaries are based on the fusion of all kinds of references.

After counting the errors of predicted gait stages, we obtained the F-score shown in Figure 9, which demonstrated the accuracy comparison based on the single reference information, fused results based on two references, and fused results based on four references for detecting gait boundaries. The single reference schemes include sagittal-axis acceleration-based references, vertical-axis acceleration-based references, sagittal-axis acceleration differential-based references, and vertical-axis acceleration differential-based references. It is shown in Figure 9 that among the four kinds of single reference information, the connection based on vertical-axis acceleration can provide more accurate swing gait phases in different walking speed scenarios, and the results based on sagittal acceleration and vertical acceleration are comparable in terms of estimation accuracy of other gait phases. The references based on the derivative of vertical and sagittal acceleration generally performed weaker than their original data in terms of accuracy, except for the push-off gait in the fast speed group only. Then we compare the results using two references with those of a single reference. It could be found that no significant improvement is obtained because the fusion results using two references are averaged values, which makes the prediction results vulnerable to the influence of the worse reference. Further, by comparing the results from using four references to the other schemes, we can find that its F-score is better than that of using two references and all single references, both in three different speed test groups and for the different gait phases.

**Figure 10:**
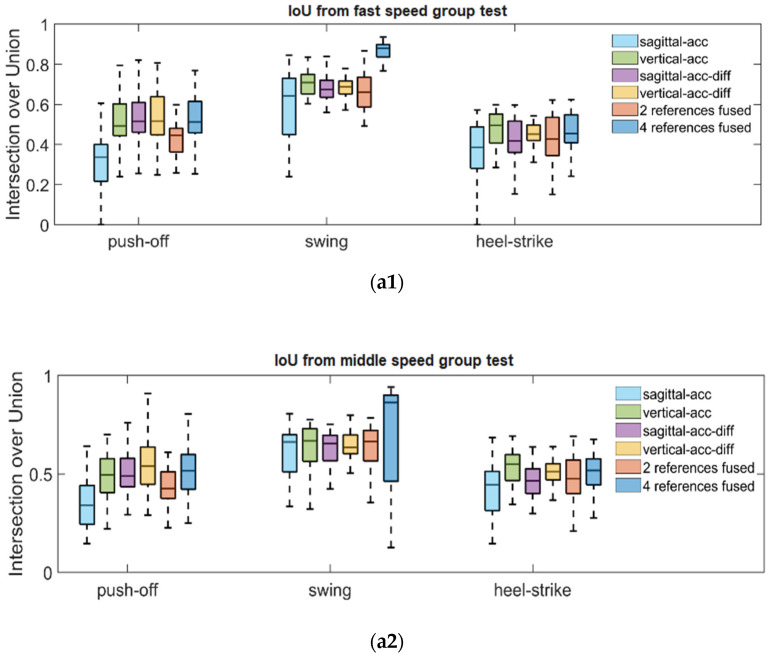

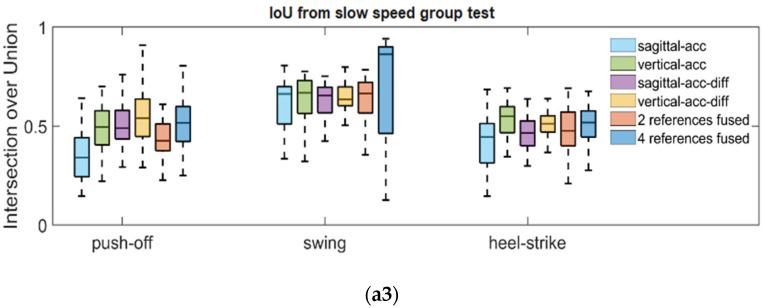
Use IoU as a metric to measure the performance of gait segmentation methods for recognizing push-off phases, swing phases, and heel-strike phases. Subfigures (**a1**–**a3**) show the F-score corresponding to three gait phases in walking with fast speed, middle speed, and slow speed, respectively.

Furthermore, Figure 10 shows the IoU corresponding to the results of using different reference schemes, demonstrating how much similarity the predicted gait segments and the real gait phases share in terms of the time range. We can find that in the gait segments that are true positives, using the vertical acceleration’s reference information gives a more consistent time range with the real gait phases than using the reference information generated from sagittal acceleration. For the gait segments predicted by using the differentiation of sagittal and vertical acceleration as single references, their IoU values were essentially comparable to that of the results supported by vertical accelerations; especially, the fast push-off predictions showed a better time range similarity with real gait push-off phases. This result indicates that proper application of differential acceleration signal can hopefully improve the temporal agreement between the predicted gait segments and their corresponding accurate gait phases. The four-reference-fusion-scheme showed better temporal similarity than the two-reference-fusion-scheme. The outputs based on the four-reference-fusion-scheme were comparable to the best results among the single references in each test group for recognizing push-off and heel-strike phases and showed the best temporal agreement for the recognizing swing gait phases.

Finally, we tested the gait segmentation algorithms with different reference configurations on full speed range walking data, and the results are shown in Table 4 and Table 5. The algorithm based on the four references achieved the best F-score on push-off and heel-strike phases and is comparable to the best F-score on swing phases based on vertical acceleration reference information. The best IoU in Table 5 is obtained for the swing phases and is comparable to the best IoUs for the push-off and heel strike phases.

In summary, we obtained the following two conclusions. Firstly, to implement gait segmentation in complex scenarios across individuals and broad velocity domains, the accuracy of gait segmentation cannot be guaranteed using only single-axis acceleration data. Moreover, the derivative of the acceleration data is more susceptible to the signal noise than the original acceleration signal. Secondly, if the process of fusing different reference information is simply calculating the average value, it might probably fail to improve the performance. Checking multiple reference items is necessary, e.g., eliminating possible outliers and fusing the selected references can lead to more accurate gait segmentation results.

### 3.3. Performance Comparison with Existing Methods for Gati Segmentation Based on Foot Movement Data

Based on the comparison results, we think the gait segmentation algorithm based on four reference information can provide accurate gait classification results in cross-individual and broad velocity domain scenarios. Thus, we consider it could serve as gait phases prediction module for subsequent step length estimation schemes.

### 3.4. Performance of Adaptive Stride Length Estimation Algorithms

We test the fusion-estimated step estimation model on a test set and compare it with the same type of model using the whole stride data as the source of features.

According to the error measurements in Table 6, we can find that the model using fusion estimation is comparable to the model using the complete stride data because the walking patterns contained in the DLR public dataset are simple and monotonous due to the constraints of the acquisition environment. Therefore, we tested the trained model in a changing speed walking scenario. It should be noted that the pre-trained model on the DLR dataset was not parameterized. Although our test scenario does not provide real-time location information, we selected a straight path and measured the distance between the beginning point and the endpoint using a laser rangefinder. One subject was asked to walk at a self-selected speed and alternate the speed based on personal choice.

After comparison in Table 7, it can be found that the fused estimation step length model based on gait phases exhibits smaller RMSE and cumulative distance relative error rates in the pedestrian variable speed walking scenario. This indicates that the gait-stage fusion-based step estimation method can demonstrate better adaptability to walking speed variation than the complete pace-based step estimation method. In addition, the proposed method in this paper still shows better accuracy without parameter customization, which shows that the gait-stage fusion-based step estimation method can meet the requirements of cross-individuals in practical applications.

## 4. Discussion

For both sagittal and vertical acceleration data, we apply the “major peak-valley pairs” to the pre-processed signals, as well as to their respective differential signals, because the differential acceleration represents the trend and rapidity of force changes on the foot, and the “major peak-valley pairs” phenomenon is also present in the differential acceleration signals at gait boundaries and other temporal locations. The pair phenomenon is also present in the acceleration differential signal. This means that the foot is subjected to the greatest forces during the short period of time at the boundary of the gait phase, while at the same time, the external forces also appear to change very dramatically in a very short period of time. Hence introducing more bases for judgment makes it easier for us to eliminate possible pseudo-gait boundaries. Furthermore, for calculating the mean value of the gait boundary based on the acceleration and its differential signal, we find that the results are much closer to the real value. Our experimental results also show that this is indeed beneficial for finding accurate gait boundaries from a practical application point of view.

**Figure 11:**
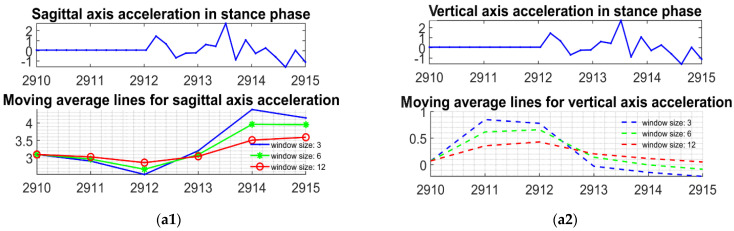

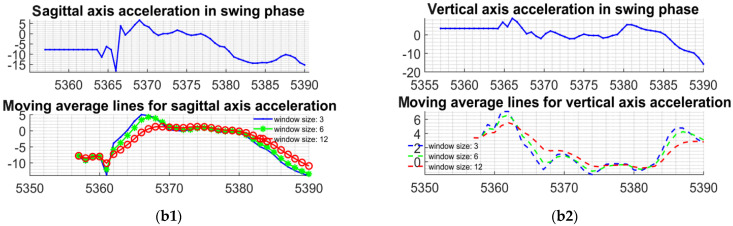
In fast and slow walking, the acceleration within the stance phase and the swing phase have comparable magnitude and similar fluctuation trends, which is the source of the stance phase identification error. The methods relying on thresholds will fail in such cases. (**a1**) Sagittal acceleration and its moving average lines in stance phase during fast walking; (**a2**) Vertical acceleration and its moving average lines in stance phase during fast walking; (**b1**) Sagittal acceleration and its moving average lines in swing phase during slow walking; (**b2**) Vertical acceleration and its moving average lines in swing phase during slow walking.

When different pedestrians walk at different speeds, the acceleration of the walking speed causes the duration of the stance phase to be significantly shorter and the fluctuation of the acceleration during the stance phase to increase. This even makes the fluctuation amplitude of the acceleration during the stance phase comparable to the fluctuation amplitude of the acceleration signal during the swing phase while the pedestrian is walking at a slow speed. One kind of this phenomenon is shown in Figure 11. We use the average of three moving, sliding windows with different scales and their sizes are three, six, and twelve, respectively. In the fast-walking scenario, the acceleration data marked as the stance phase can be seen with fluctuation intervals comparable to the fluctuation of acceleration within the swing phase of slow walking. This is an unfavorable situation for the gait segmentation algorithm because the foot is in a state of inactive force during both the stance phase and the swing phase, which leaves the sagittal-axis acceleration signal, the vertical-axis acceleration signal trending in a flat/soft state. During fast walking, we observed that the foot motion data of some subjects in the Diverse Gait Dataset also showed a certain trend during the stance phase. This is the reason why the stance phase and swing phase may be easily misidentified. We consider this a difficult problem for gait segmentation in cross-individual and wide velocity domain conditions. Thus we are exploring an adaptive thresholding method based on signal fluctuation analysis, or new features for identification and verification of easily confused stance and swing, which is one of our future works.

The fusion procedure for stride length estimation in this paper simply calculates the mean value, which is a very naive fusion method, resulting in the final step fusion results are likely to be impacted by the predicted values with large errors. Therefore, in our future work, we will explore more fusion schemes to improve the accuracy of fused step estimation based on gait information.

## 5. Conclusions

Based on the four gait labels provided by the Diverse Gait Dataset, we firstly provide a description of foot movement processes in different phases of a stride cycle from the acceleration signal perspective. We find that the switching of foot motion in different gait phases produces significant and abrupt changes in forces in the anterior-posterior direction and in the vertical direction with respect to the ground, which forms the basis of the research in this paper. Based on our understanding of the acceleration signal in gait phases, we propose a gait segmentation algorithm based on peak-valley pairs of acceleration data. Furthermore, in order to evaluate the performance of the gait segmentation method in a more detailed way, we introduce the IoU metric in the field of target detection to evaluate the coincidence of the segmented gait phases with the real gait labels in the time range. To simulate the tests in cross-individual and wide velocity domain scenarios, the proposed algorithm was evaluated on the Diverse Gait Dataset and achieved F-scores of: 0.748, 0.805, 0.819, and an IoU of: 0.487, 0.697, 0.514 for push-off, swing and heel-strike gait phases, respectively, which are better than the current well-established methods. By using the gait segmentation algorithm directly on IMU data without any label information, we find the sub-sequences of acceleration data and construct different feature sets for each gait phase, respectively. Test results show that the adaptive stride length estimation method, which is based on gait predictions fusion, show better performance at varying walking speed and can fulfill stable accuracy on different pedestrians.

## Figures and Tables

**Table 1:** A total of 22 healthy volunteers (13 males, 9 females, age 32.5 ± 7.5 years) participated in the study and were divided into different groups according to gender and height information.

Height Range (cm)	Males	Females	Number of Strides (Speed Type)		Number of Gait Phases	
155~160	-	2	fast	142	stance	487
			middle	159	pushoff	478
			slow	171	swing	474
			full speed range	472	heel-strike	474
160~165	2	3	fast	261	stance	1121
			middle	337	pushoff	1100
			slow	475	swing	1113
			full speed range	1073	heel-strike	1110
165~170	2	2	fast	298	stance	1022
			middle	324	pushoff	1013
			slow	367	swing	1113
			full speed range	989	heel-strike	998
170~176	4	1	fast	406	stance	1358
			middle	440	pushoff	1353
			slow	459	swing	1006
			full speed range	1305	heel-strike	1378
176~180	2	1	fast	123	stance	408
			middle	121	pushoff	399
			slow	146	swing	400
			full speed range	390	heel-strike	393
180~185	3	-	fast	150	stance	480
			middle	114	pushoff	470
			slow	197	swing	470
			full speed range	461	heel-strike	465

**Table 2:** Empirical models proposed for stride length estimation.

Model	Formulas (‘Step Length’ Has Been Simplified as ‘SL’)	
Weinberg	$SL=K\times \sqrt[{4}]{a_{max}-a_{min}}$	(1)
Kim	$SL=K\times \sqrt[{3}]{\frac{\sum_{i=1}^{N}\left|a_{i}\right|\;}{N}}$	(2)
Ladetto	SL=α×f+β×v+γ	(3)
Scarlett	$SL=K\times \frac{\sum_{i=1}^{N}\left|a_{i}\right|-a_{min}}{a_{max}-a_{min}}$	(4)

**Table 3:** Stride detection results for msDTW, zero-velocity based method, and SDATW in F-measure values. Best results for each speed group are highlighted in bold numbers.

Group Name (Walking Speed)	Group Volume (Strides)	msDTW	Zero-Velocity Based Method	SDATW
fast	1380	0.813	0.8128	**0.9337**
mid	1495	0.818	0.8756	**0.928**
slow	1815	0.829	0.8849	**0.9328**
all	4690	0.822	0.8592	**0.9304**

**Table 4:** Gait segmentation results for different methods in F-score values. Best results for each speed group are highlighted in bold numbers.

Gait Segmentation Method	Stance	Push-Off	Swing	Heel-Strike
FAU	0.754 ± 0.022	0.786 ± 0.048	0.84 ± 0.029	0.761 ± 0.047
WAVELET	**0.897 ± 0.018**	0.729 ± 0.132	0.735 ± 0.128	0.717 ± 0.134
PEAK-VALLEY PAIR (proposed)	0.811 ± 0.018	**0.748 ± 0.056**	**0.805 ± 0.037**	**0.819 ± 0.02**

**Table 5:** Gait segmentation results for different methods in IoU values. Best results for each speed group are highlighted in bold numbers.

Gait Segmentation Method	Stance	Push-Off	Swing	Heel-Strike
FAU	0.601 ± 0.014	0.457 ± 0.03	0.679 ± 0.026	0.345 ± 0.012
WAVELET	**0.706 ± 0.019**	0.445 ± 0.078	0.686 ± 0.075	0.341 ± 0.031
PEAK-VALLEY PAIR (proposed)	0.638 ± 0.012	**0.487 ± 0.017**	**0.697 ± 0.076**	**0.514 ± 0.01**

**Table 6:** Stride length estimation errors by using multi-gait features and full stride features.

Estimation Model		RMSE	Distance		Distance Estimation Error	
			Reference (m)	Estimated (m)	Absolute (m)	Relative (%)
Whole Stride	SVR	159.199	527.464	520.218	7.246	1.37
	SVR-Adaboost	173.922		500.011	27.453	5.2
Gait Predictions Fusion	SVR	137.917		520.137	7.327	1.39
	SVR-Adaboost	151.933		517.453	10.011	1.9

**Table 7:** Gait segmentation results for different methods in F-score values. Best results for each speed group are highlighted in bold numbers.

Estimation Model		Distance		Distance Estimation Error	
		Reference (m)	Estimated (m)	Absolute (m)	Relative (%)
Whole Stride	SVR	501.2	473.882	27.318	5.45
	SVR-Adaboost		456.749	44.451	8.87
Gait Predictions Fusion	SVR		524.671	23.471	**4.68**
	SVR-Adaboost		509.825	8.625	**1.72**
